# Hepatitis B infection reported with cancer chemotherapy: analyzing the US FDA Adverse Event Reporting System

**DOI:** 10.1002/cam4.1429

**Published:** 2018-04-16

**Authors:** Akimasa Sanagawa, Yuji Hotta, Tomoya Kataoka, Yasuhiro Maeda, Masahiro Kondo, Yoshihiro Kawade, Yoshihiro Ogawa, Ryohei Nishikawa, Masahiro Tohkin, Kazunori Kimura

**Affiliations:** ^1^ Department of Pharmacy Nagoya City University Hospital Nagoya Japan; ^2^ Department of Hospital Pharmacy Graduate School of Pharmaceutical Sciences Nagoya City University Nagoya Japan; ^3^ Department of Clinical Pharmaceutics Graduate School of Medical Sciences Nagoya City University Nagoya Japan; ^4^ Department of Regulatory Science Graduate School of Pharmaceutical Sciences Nagoya City University Nagoya Japan

**Keywords:** Cancer chemotherapy, data mining, FDA Adverse Event Reporting System, hepatitis B virus, signal detection

## Abstract

We conducted data mining using the US Food and Drug Administration (FDA) Adverse Event Reporting System (FAERS) database on spontaneously reported adverse events to evaluate the association between anticancer drug therapy and hepatitis B infection. Reports of hepatitis B infection were retrieved from the FAERS database. The reporting odds ratio (ROR) was used to estimate the association between hepatitis B infection and various anticancer agents and drug combinations. We detected statistically significant risk signals of hepatitis B for 33 of 64 anticancer agents by ROR (26 cytotoxicity drugs and seven molecular‐targeted drugs). We focused on molecular‐targeted drugs and assessed the risk of hepatitis B from specific anticancer drug combinations. The frequency of hepatitis B infection was significantly high for drugs such as rituximab, bortezomib, imatinib, and everolimus. The addition of cyclophosphamide, doxorubicin, and fludarabine to drug combinations additively enhanced the frequency of hepatitis B infection. There were no reports on hepatitis B infection associated with trastuzumab or azacitidine monotherapy. However, trastuzumab‐containing regimens (e.g., combinations with docetaxel or paclitaxel) were correlated with the incidence of hepatitis B infection, similar to azacitidine monotherapy. Our findings suggest that the concomitant use of anticancer drugs, such as trastuzumab, taxane, and azacitidine, may contribute to the risk of hepatitis B infection. The unique signals detected from the public database might provide clues to eliminate the threat of HBV in oncology.

## Introduction

In recent years, many cytotoxic and molecular‐targeted anticancer drugs have been developed, increasing the complexity of chemotherapy. Nearly 400 million people are infected with the hepatitis B virus (HBV) worldwide 1, 2 and at a risk for virus reactivation by immunosuppressive therapy for various diseases 3, 4. Chemotherapy‐induced hepatitis B reactivation is well known and can result in fulminant hepatic failure or death or both 5. Thus, prior to the initiation of immunosuppressive events (e.g., chemotherapy), HBV screening is recommended in the national and international guidelines 4, 6, 7, 8, 9. However, several cost‐effectiveness analyses have concluded that the recommended level of HBV screening should depend on the cancer type 10, 11, 12. Therefore, it is important to identify anticancer drugs associated with HBV reactivation.

Evaluating the risk of adverse events associated with anticancer polytherapy is difficult in general. A recent report recommended the creation of a public database for comprehensive and timely reporting of all drugs, either new or old, associated with HBV reactivation 13. Data mining is a useful method for identifying drugs that may induce adverse events (AEs) and is used by regulatory agencies and the pharmaceutical industry to screen drugs for further clinical review 14, 15, 16, 17, 18. The US Food and Drug Administration (FDA) Adverse Event Reporting System (FAERS) database provides a powerful means for identifying potential associations between drugs and AEs 19, 20 and exploring drug–drug interactions 21. However, there are few studies analyzing this database for anticancer agents and AEs 22, 23. In this study, we analyzed FAERS database to search for clues to eliminate the threat of HBV in oncology.

## Methods

### Data sources

Data were obtained from the FAERS website 19. Cases with missing data (drug name or adverse reactions) were excluded. For duplicate case report forms, we adopted the most recent case number 24.

### Reorganizing drug names

To accurately identify and aggregate all case reports for a marketed drug, the drug name variants (including generic names, names used outside the USA, and misspellings) were grouped under a common name. Spelling errors were detected by working pharmacists. The target drug group comprised 64 anticancer drugs searchable in the medical database DrugBank 25.

### Definition of an AE

The AEs in the FDA database were coded using preferred terms (PT) in the Medical Dictionary for Regulatory Activities (MedDRA) 26. The PT “hepatitis B” was selected as the target AE.

### Signal detection

A statistically significant association with an AE was considered a signal 20. The statistical signal strength of the association between an anticancer drug or drug combination and hepatitis B infection was calculated using the reporting odds ratio (ROR) 17 and the proportional reporting ratio (PRR) 14 as indicators. ROR has higher sensitivity than PRR. Conversely, PRR has higher specificity than ROR. ROR and PRR were calculated by identifying the case reports in datasets. A signal of the drug–event combination was detected when the lower limit of 95% confidence interval (CI) of the ROR exceeded 1. A signal was defined as a PRR of 2 or more, chi‐squared value of at least 4, and three or more cases. We added a signal count to all the cells in the corresponding 2 × 2 table. In addition, we specified cases that used target drugs listed as primary and secondary suspected drugs, and calculated the statistical signal strength.

### Evaluation of the relationship between hepatitis B infection and multidrug chemotherapy

Anticancer drugs with AE signals detected by ROR were termed “signal drugs.” Signal detection by ROR gives an estimate of relative signal strength, reflecting the frequency of a particular AE in association with a given drug, compared with other drugs for the same indication 27. Standard drug regimens for various cancers are listed in the NCCN Chemotherapy Order Templates 28. Concomitant patterns of signal drugs were determined from this list and checked by working pharmacists. The number of hepatitis B cases and all AEs was collected from concomitant patterns of signal drugs. The RORs of each combinatorial pattern for similar indications were calculated. The relationship between hepatitis B infection and multidrug chemotherapy was estimated from a comparison table of signal drug combinations. For a risk estimate of hepatitis B, the lower limit of 95% CI of ROR had to exceed 1. The analyses were conducted using SAS 9.4 (SAS Institute, Inc., Cary, NC).

## Results

### Data mining

**Figure 1:**
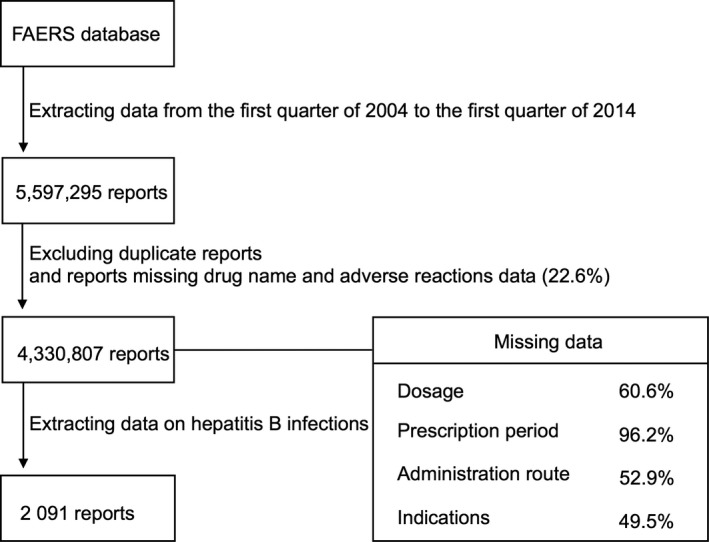
Flowchart illustrating data mining from the US Food and Drug Administration (FDA) Adverse Event Reporting System (FAERS) database.

Figure 1 shows the flowchart of data extraction. We analyzed 5,597,295 case reports received by the FDA between the first quarter of 2004 and the first quarter of 2014. After excluding duplicate reports and cases missing the drug name or adverse reaction, 4,330,807 case reports remained. The number of hepatitis B events reported during the study period was 2091. Of the hepatitis B reports, 595 (28%) lacked information on patient age. In the remaining reports, the mean age of patients with hepatitis B was 52.8 ± 16.2 years. Data on sex were missing in 228 case reports (11%); the number of male and female patients in the remaining cases was 1163 and 700, respectively.

### Anticancer drugs reported with hepatitis B events

Tables 1, 2 show the associations between chemotherapeutic drugs and reported hepatitis B events. Signals were detected for 26 of 38 cytotoxic anticancer agents and seven of 26 molecular‐targeted drugs by ROR, and for 23 of 38 cytotoxic anticancer agents, and six of 26 molecular‐targeted drugs by PRR. For dacarbazine, docetaxel (DOC), paclitaxel (PTX), and thalidomide (THAL), signals were detected only by ROR. The number of cases that used target drugs listed as primary or secondary suspected drugs, and the calculated ROR and PRR are shown in parentheses in Tables 1, 2. In the data analysis, we detected signals for busulfan and gemcitabine; however, these signals were similar to those detected for vindesine and thalidomide. It can be suggested that busulfan and gemcitabine might be associated with hepatitis B, whereas signals of vindesine and thalidomide may be specious signals. Signals of these drugs may be affected by concomitant drugs. In addition, anticancer drugs with ROR‐detected signals were classified as “signal drugs” and subjected to further analysis. We focused on combinations that included the signal‐detected molecular‐targeted drugs rituximab (Rmab), trastuzumab (Tmab), bortezomib (BOR), THAL, imatinib (GLI), everolimus (Emus), and azacitidine (AZA).

**Table 1:** Signal detection for cytotoxicity drugs associated with hepatitis B infection

	Hepatitis B events	All AEs	ROR	95% CI	PRR	*χ* ^2^
Alkylating agents						
Busulfan	5 (5)	4428 (3345)	2.34 **(3.10)**	0.97–5.64 **(1.29–7.46)**	2.34 **(3.10)**	2.61 **(5.14)**
Cyclophosphamide* ^,^ †	405 (328)	41,828 (30,046)	24.86 (26.91)	22.30–27.77 (23.91–30.30)	24.63 (26.63)	7387.94 (6803.41)
Ifosfamide* ^,^ †	17 (16)	4358 (3739)	8.17 (8.96)	5.06–13.17 (5.47–14.66)	8.14 (8.92)	98.64 (104.03)
Melphalan* ^,^ †	35 (25)	8767 (5761)	8.42 (9.08)	6.03–11.77 (6.12–13.48)	8.39 (9.05)	216.97 (168.97)
Dacarbazine*	3 (0)	1623 (1296)	3.84 (4.81)	1.24–11.92 (1.55–14.94)	3.83 **(4.80)**	3.76 **(5.62)**
Procarbazine	0 (0)	1321 (1146)				
Temozolomide* ^,^ †	15 (15)	7090 (6371)	4.41 (4.91)	2.65–7.34 (2.96–8.17)	4.41 (4.90)	35.92 (42.51)
Antimetabolites						
Cladribine* ^,^ †	6 (5)	784 (664)	16.01 (15.74)	7.16–35.78 (6.52–37.99)	15.89 (15.63)	69.34 (54.52)
Fludarabine* ^,^ †	76 (63)	11,293 (8037)	14.52 (16.83)	11.54–18.27 (13.09–21.65)	14.43 (16.71)	902.68 (887.60)
Nelarabine	0 (0)	275 (260)				
Pentostatin	1 (0)	539 (479)	3.85	0.54–27.39	3.84	0.22
Capecitabine	4 (3)	26,089 (22,494)	0.32 (0.28)	0.12–0.84 (0.09–0.85)	0.32 (0.28)	5.24 (5.02)
Cytarabine* ^,^ †	42 (34)	12,276 (9812)	7.23 (7.30)	5.33–9.82 (5.20–10.25)	7.21 (7.28)	214.21 (175.11)
Fluorouracil* ^,^ †	43 (37)	23,886 (17,266)	3.79 (4.51)	2.80–5.13 (3.26–6.24)	3.79 (4.50)	83.66 (95.57)
Gemcitabine	13 (12)	16,239 (13,614)	1.66 **(1.83)**	0.96–2.87 **(1.04–3.23)**	1.66 (1.83)	2.78 (3.71)
Methotrexate* ^,^ †	139 (90)	110,270 (24,737)	2.73 (7.85)	2.30–3.24 (6.36–9.70)	2.73 (7.83)	140.17 (506.75)
Pemetrexed	2 (2)	7091 (6438)	0.59 (0.64)	0.15–2.34 (0.16–2.57)	0.58 (0.64)	0.25 (0.12)
Antitumor antibiotics						
Bleomycin* ^,^ †	13 (12)	3035 (2508)	8.96 (10.00)	5.19–15.47 (5.66–17.67)	8.92 (9.96)	83.193 (87.52)
Dactinomycin* ^,^ †	5 (4)	1033 (905)	10.09 (9.21)	4.19–24.32 (3.44–24.61)	10.05 (9.17)	32.12 (21.49)
Mitomycin	0 (0)	1305 (1070)				
Daunorubicin* ^,^ †	6 (6)	4190 (3296)	2.97 (3.78)	1.33–6.63 (1.70–8.44)	2.97 (3.78)	5.99 (9.61)
Doxorubicin* ^,^ †	293 (240)	28,552 (22,536)	24.80 (25.04)	21.91–28.07 (21.88–28.67)	24.56 (24.79)	5675.18 (4830.97)
Epirubicin* ^,^ †	28 (24)	5817 (4578)	10.14 (11.02)	6.97–14.73 (7.36–16.50)	10.09 (10.97)	217.47 (205.37)
Idarubicin* ^,^ †	7 (7)	1718 (1278)	8.49 (11.44)	4.04–17.87 (5.43–24.07)	8.46 (11.38)	38.80 (56.13)
Mitoxantrone* ^,^ †	26 (24)	3357 (2589)	16.35 (19.58)	11.09–24.11 (13.07–29.34)	16.23 (19.41)	352.25 (396.47)
Asparaginase	4 (4)	3633 (3249)	2.28 (2.55)	0.86–6.10 (0.96–6.82)	2.28 (2.55)	1.74 (2.38)
Antimicrotubule agents						
Vinblastine* ^,^ †	6 (5)	1552 (1177)	8.06 (8.85)	3.61–17.98 (3.67–21.33)	8.03 (8.85)	30.14 (27.22)
Vincristine* ^,^ †	288 (228)	20,500 (15,484)	34.05 (34.60)	30.05–38.59 (30.13–39.74)	33.59 (34.11)	7826.67 (6501.79)
Vindesine* ^,^ †	3 (1)	472 (344)	13.26 **(6.04)**	4.26–41.30 **(0.85–43.01)**	13.18 **(6.02)**	22.67 **(0.67)**
Vinorelbine	1 (0)	4595 (3152)	0.45	0.06–3.20	0.45	0.23
Eribulin	0 (0)	678 (613)				
Docetaxel*	17 (13)	18,227 (14,161)	1.94 (1.91)	1.20–3.13 (1.11–3.29)	1.94 (1.91)	6.77 (4.71)
Paclitaxel*	23 (20)	24,039 (19,372)	1.99 (2.15)	1.32–3.01 (1.38–3.34)	1.99 **(2.15)**	10.29 **(11.06)**
Topoisomerase inhibitors						
Irinotecan* ^,^ †	20 (18)	11,671 (8802)	3.58 (4.27)	2.30–5.56 (2.68–6.79)	3.57 (4.27)	34.22 (41.42)
Etoposide* ^,^ †	71 (58)	14,231 (11,331)	10.71 (10.93)	8.45–13.58 (8.41–14.19)	10.66 (10.88)	591.46 (496.34)
Platinum‐based agents						
Carboplatin* ^,^ †	24 (19)	21,457 (16,499)	2.33 (2.40)	1.56–3.49 (1.53–3.77)	2.33 (2.40)	16.76 (13.99)
Cisplatin* ^,^ †	36 (34)	22,054 (17,654)	3.43 (4.04)	2.46–4.77 (2.88–5.68)	3.42 (4.04)	58.33 (73.52)
Oxaliplatin	13 (8)	17,247 (14,114)	1.57 (1.17)	0.91–2.70 (0.59–2.35)	1.57 (1.18)	2.10 (0.07)

*Signal detected by ROR; ^†^Signal detected by PRR.

The numbers in parentheses: target drugs were listed as primary suspected drug or secondary suspected drug. Bold, change of interpretation of signal detection. AE, adverse event; ROR, reporting odds ratio; PRR, proportional reporting ratio; CI, confidence interval.

**Table 2:** Signal detection for molecular‐targeted drugs associated with hepatitis B infection

	Hepatitis B events	All AEs	ROR	95% CI	PRR	*χ* ^2^
Rituximab* ^,^ †	445 (427)	33,225 (28,845)	35.43 (38.83)	31.89–39.36 (34.89–43.21)	34.97 (38.27)	11,537.76 (12,309.87)
Trastuzumab* ^,^ †	15 (13)	12,113 (10,310)	2.58 (2.62)	1.55–4.29 (1.52–4.53)	2.58 (2.62)	12.84 (11.40)
Gemtuzumab ozogamicin	2 (1)	1766 (1700)	2.35 (1.22)	0.59–9.40 (0.17–8.66)	2.35 (1.22)	0.49 (0.13)
Bevacizumab	15 (15)	38,038 (35,757)	0.82 (0.86)	0.49–1.36 (0.52–1.44)	0.82 (0.87)	0.45 (0.18)
Cetuximab	3 (3)	14,789 (14,304)	0.42 (0.43)	0.14–1.30 (0.14–1.35)	0.42 (0.43)	1.86 (1.69)
Panitumumab	0 (0)	3101 (3016)				
Ibritumomab tiuxetan	0 (0)	124 (115)				
Gefitinib	1 (1)	3924 (3761)	0.53 (0.55)	0.07–3.75 (0.08–3.91)	0.53 (0.55)	0.08 (0.06)
Imatinib* ^,^ †	31 (31)	19,945 (19,333)	3.26 (3.36)	2.28–4.64 (2.36–4.79)	3.25 (3.36)	45.46 (48.23)
Bortezomib* ^,^ †	57 (54)	16,693 (13,373)	7.26 (8.59)	5.58–9.46 (6.55–11.26)	7.24 (8.56)	292.40 (343.98)
Erlotinib	1 (1)	23,275 (22,676)	0.09 (0.09)	0.01–0.63 (0.01–0.65)	0.09 (0.09)	8.49 (8.20)
Crizotinib	0 (0)	2153 (2133)				
Sorafenib	9 (7)	10,944 (10,485)	1.71 (1.38)	0.89–3.29 (0.66–2.91)	1.71 (1.38)	1.96 (0.41)
Sunitinib	7 (7)	17,207 (16,569)	0.84 (0.87)	0.40–1.77 (0.42–1.84)	0.84 (0.88)	0.08 (0.03)
Axitinib	0 (0)	1900 (1867)				
Pazopanib	0 (0)	4860 (4794)				
Nilotinib	4 (4)	6414 (6300)	1.29 (1.32)	0.48–3.45 (0.49–3.51)	1.29 (1.32)	0.05 (0.07)
Dasatinib	1 (1)	5280 (5033)	0.39 (0.41)	0.06–2.78 (0.06–2.92)	0.39 (0.41)	0.43 (0.36)
Lapatinib	6 (5)	8938 (8537)	1.39 (1.21)	0.62–3.10 (0.50–2.92)	1.39 (1.21)	0.33 (0.04)
Everolimus* ^,^ †	10 (10)	8370 (7989)	2.48 (2.60)	1.33–4.62 (1.40–4.85)	2.48 (2.60)	7.39 (8.27)
Thalidomide*	15 (13)	18,373 (16,889)	1.70 **(1.60)**	1.02–2.82 **(0.93–2.76)**	1.70 (1.60)	3.59 (2.32)
Lenalidomide	23 (19)	51,724 (50,904)	0.92 (0.77)	0.61–1.39 (0.49–1.21)	0.92 (0.77)	0.09 (1.06)
Temsirolimus	2 (2)	2905 (2802)	1.43 (1.48)	0.36–5.71 (0.37–5.92)	1.43 (1.48)	0.01 (0.02)
Vorinostat	0 (0)	1183 (1122)				
Afatinib	0 (0)	276 (272)				
Azacitidine* ^,^ †	6 (6)	4393 (4048)	2.84 (3.08)	1.27–6.33 (1.38–6.87)	2.38 (3.08)	5.39 (6.44)

*Signal detected by ROR; ^†^Signal detected by PRR.

The numbers in parentheses: target drugs were listed as primary suspected drug or secondary suspected drug. Bold, change of interpretation of signal detection. AE, adverse event; ROR, reporting odds ratio; PRR, proportional reporting ratio; CI, confidence interval.

### Hepatitis B reported with Rmab‐based chemotherapy for lymphoma

**Figure 2:**
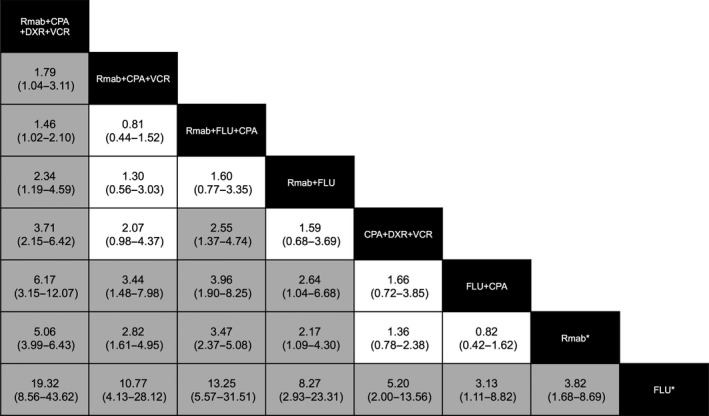
Estimates of hepatitis B virus infection risk in chemotherapy regimens for lymphoma. Chemotherapeutic drug combinations are listed in black cells. The estimate is located at the intersection of the column‐defined combination pattern and the row‐defined combination pattern. To obtain reporting odds ratios (RORs) for comparisons in the opposing direction, the reciprocals should be taken. For hepatitis B infection risk, an ROR value below 1 favors the column‐defined combination pattern. Statistically significant estimates appear in gray cells. The numbers in the parentheses indicate 95% confidence intervals. The corresponding data are listed in Table 3.

Table 3 outlines the drug combinations reported for lymphoma treatment and the associated number of AE reports. Figure 2 shows the frequency of hepatitis B reported among lymphoma treatment groups; RORs were calculated from the data in Table 3, and not all combination patterns were included. However, of the 445 hepatitis B cases, 350 (78.7%) were reported with Rmab‐containing treatments.

**Table 3:** Drug combinations and adverse events reported for lymphoma

	Hepatitis B events	All adverse events
Rmab+CPA+DXR+VCR	182	5313
Rmab+CPA+VCR	14	722
Rmab+FLU+CPA	36	1516
Rmab+FLU	9	602
Rmab^a^	109	15,668
FLU+CPA	9	1574
FLU^a^	6	3274

a Only one signal drug.

Rmab, rituximab; CPA, cyclophosphamide; DXR, doxorubicin; VCR, vincristine; FLU, fludarabine.

The combination Rmab+cyclophosphamide (CPA)+doxorubicin (DXR)+vincristine (VCR) exhibited a substantially higher relevance to hepatitis B infection than other combinations. All lower limits of 95% CIs in the RORs of this drug combination in the column exceeded 1 (Fig. 2). Conversely, fludarabine (FLU) exhibited a substantially lower relevance to hepatitis B infection than other combinations; all lower limits of the 95% CIs in the row exceeded 1. Hepatitis B infection of all other drug combinations involving Rmab was reported at a higher frequency than FLU+CPA, Rmab monotherapy, and FLU monotherapy. The contribution of Rmab+FLU+CPA combination to hepatitis B infection was reported at a higher frequency than that of CPA+DXR+VCR combination. These results indicate that a specific combination may contribute more to hepatitis B infection. The combination most associated with hepatitis B infection was Rmab+CPA+DXR+VCR.

### Hepatitis B reported with Tmab‐based chemotherapy for breast cancer

**Figure 3:**
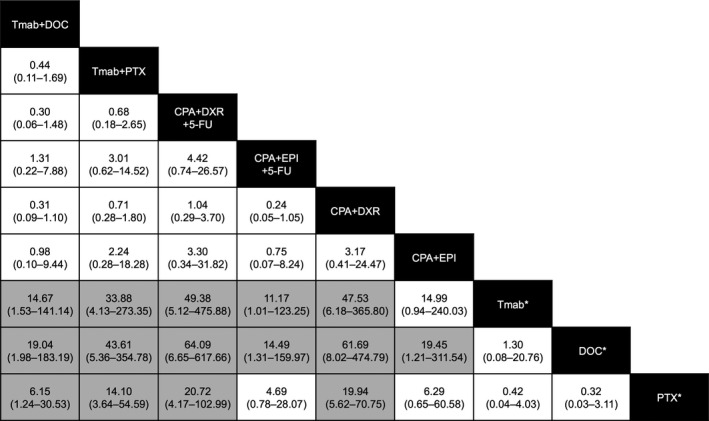
Estimates of hepatitis B infection risk in chemotherapy regimens for breast cancer. Chemotherapeutic drug combinations are listed in black cells. The estimate is located at the intersection of the column‐defined combination pattern and the row‐defined combination pattern. To obtain reporting odds ratios (RORs) for comparisons in the opposing direction, the reciprocals should be taken. For hepatitis B infection risk, an ROR value below 1 favors the column‐defined combination pattern. Statistically significant estimates appear in gray cells. The numbers in the parentheses indicate 95% confidence intervals. The corresponding data are listed in Table 4.

Table 4 outlines the drug combinations reported for breast cancer treatment and the associated number of AE reports. Of the 15 hepatitis B cases, 11 (73.3%) were reported with Tmab‐containing regimens. Figure 3 shows the frequency of hepatitis B reported among breast cancer treatment groups. The frequency of hepatitis B reported for Tmab+DOC and Tmab+PTX was higher than that for Tmab monotherapy, DOC monotherapy, and PTX monotherapy. The frequency of reporting of hepatitis B for CPA+DXR+fluorouracil (5‐FU) and CPA+DXR was higher than that for the three monotherapies, whereas that of CPA+epirubicin (EPI)+5‐FU was higher than that of Tmab and DOC monotherapies. Similarly, the frequency of reporting of hepatitis B for CPA+EPI combination was higher than that of DOC monotherapy.

**Table 4:** Drug combinations and adverse events reported for breast cancer

	Hepatitis B events	All adverse events
Tmab+DOC	3	1232
Tmab+PTX	7	1255
Tmab^a^	1	6024
DOC^a^	1	7819
PTX^a^	3	7582
CPA+DXR+5‐FU	3	366
CPA+EPI+5‐FU	2	1079
CPA+DXR	12	1533
CPA+EPI	1	402

a Only one signal drug.

Tmab, trastuzumab; DOC, docetaxel; PTX, paclitaxel; CPA, cyclophosphamide; DXR, doxorubicin; 5‐FU, fluorouracil; EPI, epirubicin.

### Hepatitis B reported with BOR‐ or THAL‐based chemotherapy for multiple myeloma (MM)

**Figure 4:**
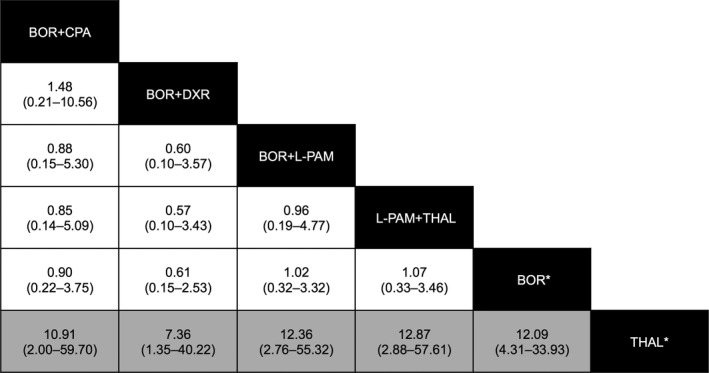
Estimates of hepatitis B infection risk in chemotherapy regimens for multiple myeloma. Chemotherapeutic drug combinations are listed in black cells. The estimate is located at the intersection of the column‐defined combination pattern and the row‐defined combination pattern. To obtain reporting odds ratios (RORs) for comparisons in the opposing direction, reciprocals should be taken. For hepatitis B infection risk, a ROR value below 1 favors the column‐defined combination pattern. Statistically significant estimates appear in gray cells. The numbers in the parentheses indicate 95% confidence intervals. The corresponding data are listed in Table 5.

Table 5 outlines the drug combinations reported for MM treatment and the associated number of AE reports. Of the 57 hepatitis B cases, 44 (77.2%) were reported with BOR‐containing treatments. Figure 4 shows the reporting frequency of hepatitis B among MM treatment groups. The reporting frequency of hepatitis B for combination therapy with BOR did not exceed the frequency for BOR monotherapy. The frequency of reporting of hepatitis B for THAL was substantially lower than that of BOR monotherapy or any drug combination, except for BOR+THAL.

**Table 5:** Drug combinations and adverse events reported for multiple myeloma

	Hepatitis B events	All adverse events
BOR+CPA	2	631
BOR+DXR	2	935
BOR+L‐PAM	3	836
BOR+THAL	0	802
L‐PAM+THAL	3	803
BOR^a^	37	10,504
THAL^a^	4	13,729

a Only one signal drug.

BOR, bortezomib; L‐PAM, melphalan; THAL, thalidomide, CPA, cyclophosphamide; DXR, doxorubicin.

### Hepatitis B reported with GLI‐, Emus‐, or AZA‐based chemotherapy

Table 6 shows that the prevalence of hepatitis B and other AEs calculated for GLI, Emus, and AZA monotherapies did not vary substantially from the prevalence calculated for drug combinations that included these drugs. This implies that these drugs were not used with other signal drugs and reveals that the use of these three drugs or their indications is associated with hepatitis B.

**Table 6:** Comparison of adverse events in monotherapies and in combinations that did not include other signal drugs

	Hepatitis B events	All adverse events
GLI monotherapy	31	19,945
GLI^a^	31	18,983
Emus monotherapy	10	8370
Emus^a^	9	7710
AZA monotherapy	6	4393
AZA^a^	5	4051

a The chemotherapy regimen included other drugs, but none were signal drugs.

GLI, imatinib; Emus, everolimus; AZA, azacitidine.

## Discussion

We identified anticancer drugs associated with hepatitis B infection by conducting a comprehensive signal‐detection analysis. First, we used the PRR method to distinguish true positives from pseudo positives. However, in the PRR method, risk signals may be missed because the interpretation of signals is convoluted for AEs associated with cancer chemotherapy. Meanwhile, there is no gold standard signal‐detection method. The ROR method probably detected pseudopositives owing to its high sensitivity and potential signal–signal interactions. Therefore, we used ROR to avoid overlooking signals. The number of signals detected by ROR slightly exceeded the number detected by PRR. Some of the signal drugs detected by ROR have been reported to be associated with hepatitis B 29, 30. Hence, we performed sensitivity analysis. However, these signal drugs are not necessarily used alone in clinical settings. In cancer chemotherapy, several anticancer drugs are frequently combined. To our knowledge, no report has been published on the association between multiple‐drug anticancer therapy and risk signals in large‐scale postmarketing databases for adverse drug reactions to date. We focused on signal‐detected molecular‐targeted drug‐containing combinations, because indications for molecular‐targeted drugs are limited compared with indications for cytotoxic anticancer drugs. Tables 3, 4, 5 show that these combinations reasonably account for most hepatitis B events reported for patients receiving molecular‐targeted drugs. Cytotoxic anticancer drugs are administered in multiple combination patterns, which are difficult to track.

Rmab is well known as a drug associated with HBV reactivation 3, 4, 11, 13, 29, 30. Among signal drugs, Rmab has the highest ROR score. Rmab is frequently used with CPA, DXR, and VCR to treat lymphoma. The drug combination R‐CHOP (Rmab, CPA, DXR, VCR, and prednisone) is a typical regimen for lymphoma. FLU is another drug used to treat lymphoma. The ROR scores of CPA, DXR, VCR, and FLU were relatively high among the signal drugs. We suspected that Rmab affected the ROR scores of the other signal drugs. First, we analyzed the drug combinations. Second, cases that involved target signal drugs only were regarded as patients receiving multiple‐drug anticancer combination therapy. We attempted to identify key drugs associated with hepatitis B infection by subtracting the signal drugs. Signal–signal interactions and differences in risk have not been reported so far.

Tmab and combined‐drug regimens containing DXR, CPA, EPI, or 5‐FU are often used to treat breast cancer. Although a relationship between anthracycline and hepatitis B has been reported, Tmab treatment has not been previously linked to hepatitis B 29, 30. DOC and PTX are frequently used in conjunction with Tmab. We found that combinations of Tmab and taxane are likely to contribute to hepatitis B infection, whereas Tmab monotherapy may not. PRR did not detect signals for DOC and PTX and may lead to overlooking true positives. The addition of 5‐FU to drug combinations may not contribute to hepatitis B risk.

Bortezomib and thalidomide are used in treating MM. Several cases of hepatitis B infection associated with BOR have been reported 31, 32, 33. CPA, DXR, and melphalan (L‐PAM) are also used in the treatment of MM, and lenalidomide may be indicated for MM. However, we did not detect a signal for lenalidomide. We found that all drug monotherapies and polytherapies for MM conferred similar risks of hepatitis B infection, with the exception of THAL monotherapy. THAL signals were influenced by other signal drugs. PRR did not detect signals for THAL. Immunomodulatory drugs such as THAL may not pose a risk for hepatitis B infection in MM.

A few cases were treated with GLI, Emus, or AZA and other signal drugs, although these drugs are usually administered alone. This suggests that the signal detected for these drugs was dependent on the target drug itself. GLI and Emus have been reported to exacerbate HBV 34, 35, whereas little is known about the risk of hepatitis B associated with AZA. AZA has been shown to increase the production of hepatitis B surface antigen in vitro 36.

As mentioned above, we found a risk of hepatitis B infection in treatments containing AZA or Tmab and taxane combinations and showed that signals detected for anticancer drugs are strongly affected by combinational drug therapy. In addition, hepatitis B risk appears to increase additively by concomitant use of some key anticancer drugs. The FAERS database is considered a valuable tool; however, the following limitations inherent to spontaneous reporting have been pointed out 17: duplicate records, missing data, misspelling of drug names, under‐reporting, over‐reporting for drugs involved in safety alerts, and reporting rate on the length of time. Reporting bias has been discussed in pharmacovigilance of oncology drugs 37. We minimized biases introduced by some limitations as much as possible through data cleaning. However, biases such as under‐reporting and reporting rate may affect the number of cases reported. The existence of indication bias may also affect the results of this study.

We must state that the PT “hepatitis B” includes lowest level terms (LLT) such as serum hepatitis, hepatitis B reactivation, hepatitis B flare, hepatitis B aggravated, viral hepatitis B, hepatitis homologous serum‐like, and HBV coinfection. From a clinical oncology standpoint, we assumed almost all hepatitis B cases that used anticancer drugs as hepatitis B reactivation or flare cases. However, analyses are generally performed at the PT level, and analyses at the LLT level should be avoided. Incidentally, the level of terms may be changed by a change request from users. In MedDRA ver.20.0 (2017 March), the term level of hepatitis B reactivation was changed from LLT to PT. Therefore, hepatitis B reactivation should be analyzed at the PT level in a voluntary reporting system. The influence of non‐anticancer drugs used concomitantly was not analyzed; these drugs may affect hepatitis B reactivation. Nuclear analogs, for instance, are known to contribute to the prevention of HBV reactivation 38. Conversely, HBV replication increases in the presence of glucocorticoid agents 39. However, drugs used to treat cancer symptoms or the side effects of treatment were not specified in our FAERS searches. Hematopoietic stem cell transplantation may contribute to the risk of hepatitis B infection in lymphoma and MM 40, 41. The prevalence of HBV infection varies across countries, regions, and institutions 6. However, geographical data were not reported in FAERS.

Owing to the above limitations, the approaches using voluntary reporting systems cannot replace traditional methods. However, it is difficult to detect hepatitis B associated with cancer chemotherapy by randomized clinical trials. Conversely, the postmarketing spontaneous reporting system can generate alert signals that may not be detected in clinical trials 42. The list of potential signals of serious risks/new safety information identified from FAERS has been published 19. We cannot avoid paying attention to the information about potential signals from each regulatory authority.

Our analytical method may offer a way to estimate HBV infection risk in cancer therapy. Large population‐based studies would be required to validate our risk estimates. However, FAERS is the largest repository of spontaneously reported adverse events in the world. In the analysis of rare AEs, its sensitivity is limited by the number of events reported. However, we were not able to avoid the problem of sample size and signal‐detection methods as mentioned above. We expected to find novel signals by continual surveillance using the database of each regulatory authority. Recently, it was reported that the drug–drug interactions detected from FAERS were determined by fundamental research using animal models 43. The approaches using cultured cells have also been reported 44, 45. Hence, not only epidemiological approaches but also fundamental research may be important.

In actual innovations, it is possible that the approach of this study may apply to the other rare AEs associated with cancer chemotherapy. We hope that this study based on public databases can promote researches on rare AEs associated with cancer chemotherapy and eventually affect clinical decisions or guidelines or both.

## Conflict of Interest

The authors declare no conflict of interests.
